# Linking Aortic Mechanical Properties, Gene Expression and Microstructure: A New Perspective on Regional Weakening in Abdominal Aortic Aneurysms

**DOI:** 10.3389/fcvm.2021.631790

**Published:** 2021-02-15

**Authors:** Arianna Forneris, Jacob Kennard, Alina Ismaguilova, Robert D. Shepherd, Deborah Studer, Amy Bromley, Randy D. Moore, Kristina D. Rinker, Elena S. Di Martino

**Affiliations:** ^1^Biomedical Engineering, University of Calgary, Calgary, AB, Canada; ^2^Department of Civil Engineering, University of Calgary, Calgary, AB, Canada; ^3^Department of Pathology and Laboratory Medicine, University of Calgary, Calgary, AB, Canada; ^4^Department of Surgery, University of Calgary, Calgary, AB, Canada; ^5^Department of Chemical and Petroleum Engineering, University of Calgary, Calgary, AB, Canada; ^6^Department of Physiology and Pharmacology, University of Calgary, Calgary, AB, Canada

**Keywords:** Abdominal Aortic Aneurysms, RNA, Computational Fluid Dynamics, thrombus, *in vivo* strain

## Abstract

**Background:** Current clinical practice for the assessment of abdominal aortic aneurysms (AAA) is based on vessel diameter and does not account for the multifactorial, heterogeneous remodeling that results in the regional weakening of the aortic wall leading to aortic growth and rupture. The present study was conducted to determine correlations between a novel non-invasive surrogate measure of regional aortic weakening and the results from invasive analyses performed on corresponding *ex vivo* aortic samples. Tissue samples were evaluated to classify local wall weakening and the likelihood of further degeneration based on non-invasive indices.

**Methods:** A combined, image-based fluid dynamic and *in-vivo* strain analysis approach was used to estimate the Regional Aortic Weakness (RAW) index and assess individual aortas of AAA patients prior to elective surgery. Nine patients were treated with complete aortic resection allowing the systematic collection of tissue samples that were used to determine regional aortic mechanics, microstructure and gene expression by means of mechanical testing, microscopy and transcriptomic analyses.

**Results:** The RAW index was significantly higher for samples exhibiting lower mechanical strength (*p* = 0.035) and samples classified as low elastin content (*p* = 0.020). Samples with higher RAW index had the greatest number of genes differentially expressed compared to any constitutive metric. High RAW samples showed a decrease in gene expression for elastin and a down-regulation of pathways responsible for cell movement, reorganization of cytoskeleton, and angiogenesis.

**Conclusions:** This work describes the first AAA index free of assumptions for material properties and accounting for patient-specific mechanical behavior in relation to aneurysm strength. Use of the RAW index captured biomechanical changes linked to the weakening of the aorta and revealed changes in microstructure and gene expression. This approach has the potential to provide an improved tool to aid clinical decision-making in the management of aortic pathology.

## Introduction

Current clinical practice for assessing the risk of abdominal aortic aneurysm (AAA) rupture relies on the vessel's maximal transverse diameter, as well as aneurysm expansion rate, measured through either ultrasound or computed tomography (CT). This method is simplistic and does not account for the intra subject heterogeneity of AAAs, leading to cases of misdiagnosed rupture risk, and a rupture rate that exceeds 2% per year in populations considered to have stable aneurysms (1–3). Moreover, elective surgical treatments (both open repairs and minimally invasive endovascular procedures) carry their own risks and complications and should be reserved to those cases where the risk of sudden aortic rupture outweighs the surgical risks. Therefore, it is crucial to define clinical guidelines that allow for a reliable and accurate estimate of the rupture potential for individual aortas. This work represents the first report of an AAA index free of assumptions for tissue material properties and accounting for patient specific mechanical behavior in relation to aneurysm strength.

From a mechanical perspective, aortic rupture likely occurs when the stress at the wall exceeds the strength of the arterial tissue. Moreover, aneurysm initiation and progression are multifactorial processes that lead to the weakening of the aortic wall at a local level. Hemodynamic factors are involved in signaling pathways and the activation of mechanosensitive receptors that mediate pathological wall remodeling and mechanical weakening. Previous studies have linked aortic expansion and rupture to regions of disturbed hemodynamics characterized by low wall-shear stress, and thick intraluminal thrombus (ILT) deposition (4–6). Such investigations, however, failed to provide a clear information on the state of regional weakening of the aortic wall.

It is well-understood that as AAA disease progresses, the aortic wall undergoes progressive heterogeneous remodeling leading to local structural changes and an altered biomechanics (7, 8). In order to link structural and mechanical changes to disease progression, previous studies often focused on *ex vivo* analysis of a representative aneurysmal tissue sample, disregarding the importance of the heterogeneous remodeling found in individual aneurysms. In this context, the deformability of the aortic wall appears to be related to its strength; therefore, areas at elevated strain may indicate structural weakening providing a rationale for non-invasive localized wall strength evaluation (9). Biomechanics-based indices, aimed at estimating the rupture risk of individual aortas, have been proposed over the past decades, showing the potential of this approach (10, 11). These studies, however, relied on finite element analysis for the estimation of local wall stress, and required assumptions for material properties for both the aortic wall and the ILT. *In vivo* three-dimensional principal strain analysis, on the other hand, is performed directly on dynamic images and does not require constitutive model assumptions (12–14).

Based on this information, a previous case study from our group proposed a combined fluid dynamic and *in vivo* strain analysis approach to develop a new index that proved able to identify the impending rupture location for a case of AAA, showing the potential of combined analysis to help aortic assessment (15).

In terms of transcriptomic analysis, literature findings point to the increased expression of genes responsible for pathways of extracellular matrix (ECM) degradation in aneurysms (16). These studies, however, do not account for the heterogeneity in aneurysmal tissue and thus do not find strong evidence of changes in gene expression linked to the progression of AAA.

The present study investigated a novel index, Regional Aortic Weakness (RAW), to assess a cohort of AAA patients prior to elective repair to correlate this non-invasive surrogate measure of regional weakening with results from mechanical testing and transcriptomic and microscopy analyses performed on corresponding *ex vivo* aortic samples. This approach has the potential to provide a new classifier (the RAW index) and enable exploration of the local biological changes that result in the regional weakening of the aortic wall and the progression of the aneurysmal disease.

## Methods

**Figure 1:**
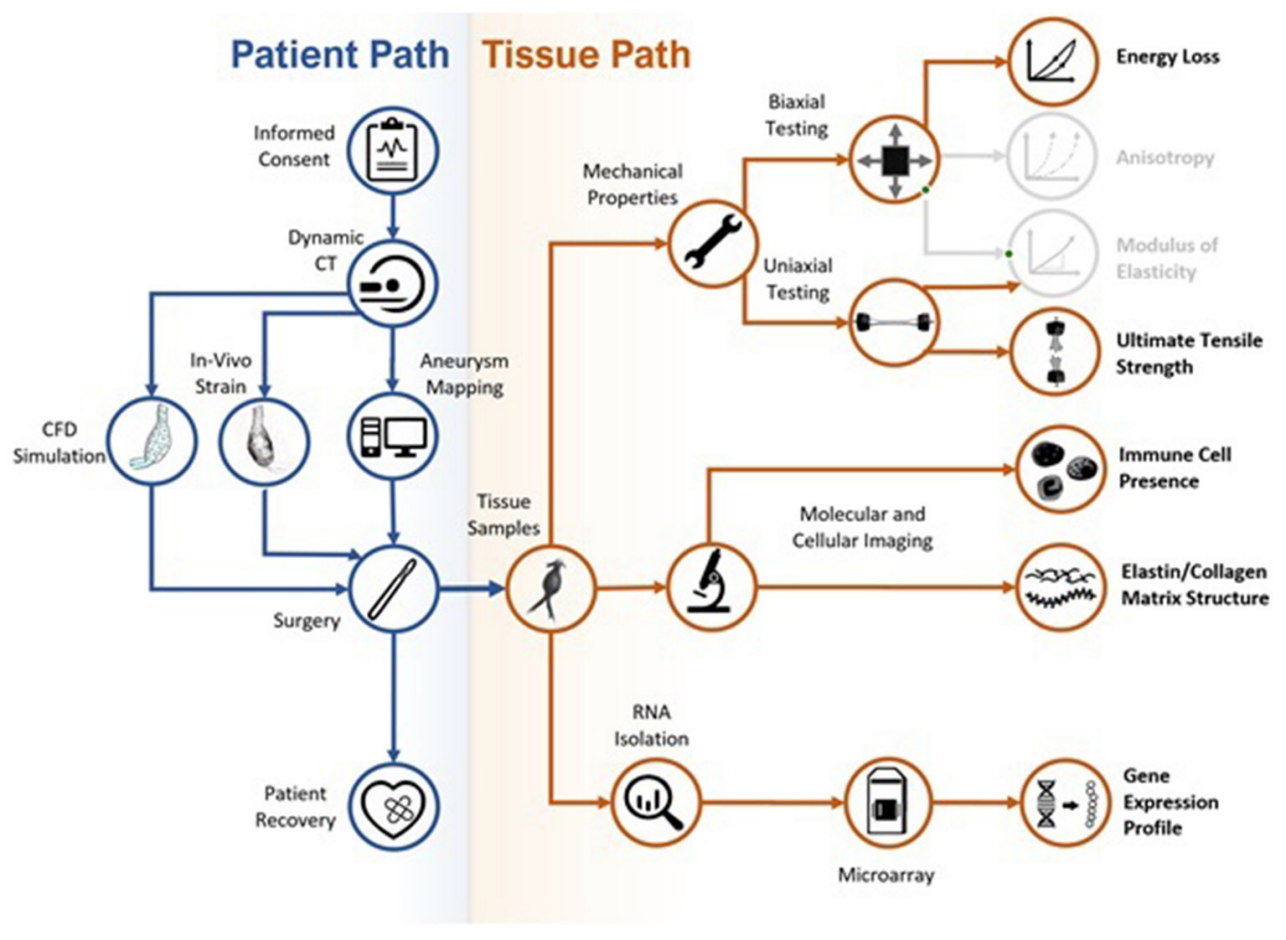
Flow chart of the study workflow. Colored sections are presented in the current paper; gray sections represent data that was collected during the study but are not presented in the current paper.

A complete study workflow is shown in Figure 1. The research protocol was approved by the University of Calgary Conjoint Health Research Ethics Board (CHREB–Ethics ID #REB15-0777), and patients' informed written consent was obtained prior to study enrollment and data collection. The study focused on a population of AAA patients that consented to participate in the approved protocol, including pre-operative electrocardiography-gated dynamic CT imaging, between 2016 and 2019.

**Figure 2:**
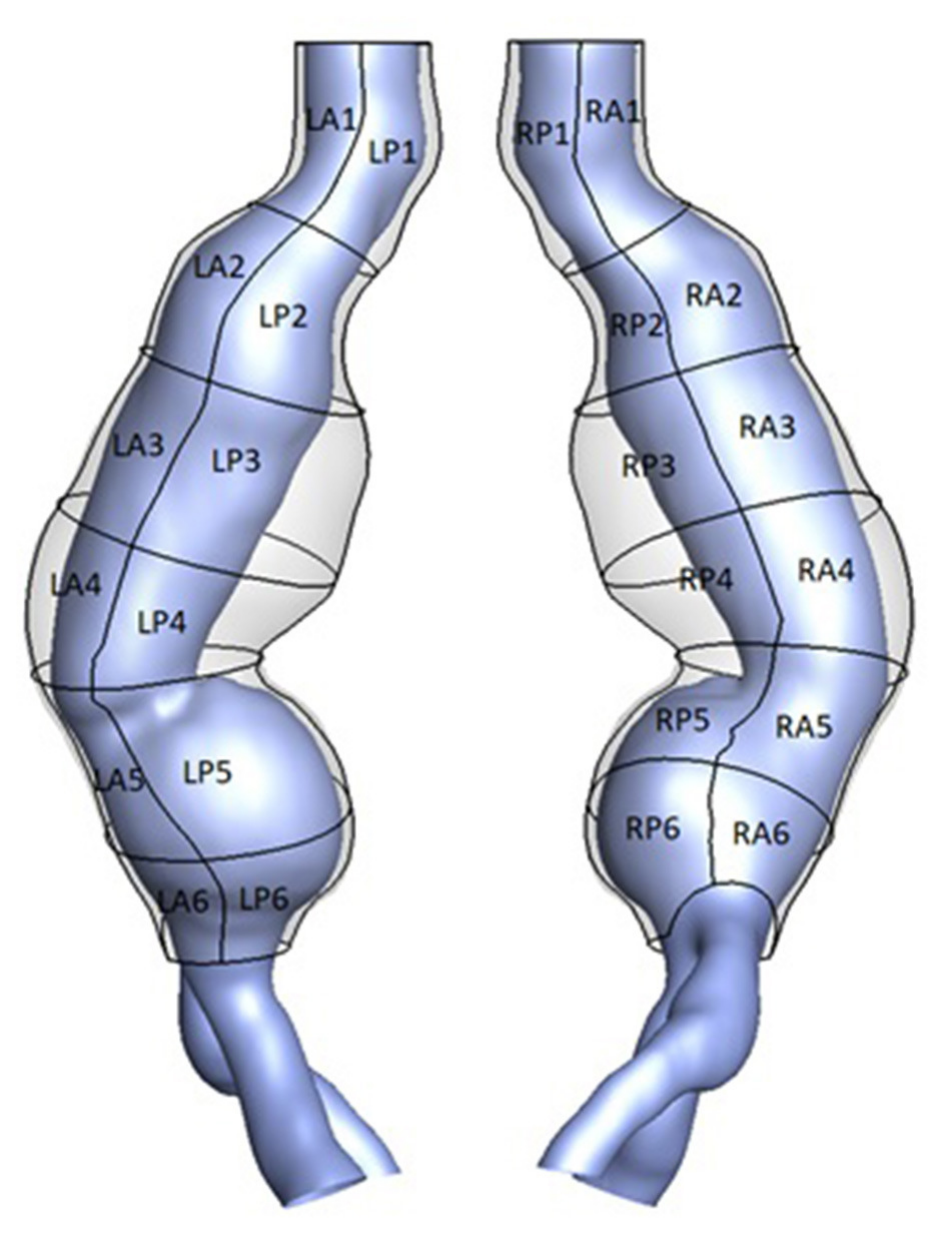
3D geometry of the outer aortic wall (transparent) and lumen (purple) for an example AAA case. A patching system defined 24 patches based on location. LP denotes “left posterior,” RA denotes “right anterior”.

The commercial imaging software Simpleware ScanIP (Synopsys, US) was used for image processing and segmentation of the aortic lumen and outer wall from CT scans of individual patients. A patching system defined 24 patches by sectioning the aortic geometry (both lumen and outer wall) perpendicularly to the lumen centerline (Figure 2). The patching served as sampling grid during tissue collection in the surgery room enabling the tracking of samples location on the aortic wall as well as statistical analysis on aortic regions.

Each analysis, namely the *in vivo* analysis to obtain the RAW index, mechanical testing, microscopy analysis and transcriptomic analysis, was performed independently by different researchers. In order to prevent biases, the RAW index for each aortic region was not made available to other researchers until completion of their analyses.

### ILT Thickness

The two reconstructed geometries representing the aortic lumen and outer aortic wall for each patient were imported in Icem (Ansys, US) for discretization into triangular shell elements. ILT thickness was measured with an in-house Matlab (MathWorks, US) algorithm as the average distance between each mesh point at the AAA outer wall and its neighbor points at the lumen surface within a specified radius.

### Computational Fluid Dynamic

Sensitivity analysis was performed to select the most appropriate volumetric mesh size with a final mesh density ranging between 2 and 3.5 million elements among the different geometries. Unsteady CFD simulations were performed in Fluent (Ansys, US) by employing a semi-implicit method for pressure linked equations (SIMPLE) algorithm for pressure-velocity coupling and a second order implicit transient formulation. An inflow velocity boundary condition was prescribed at the inlet of the fluid domain based on flow rate in the descending aorta (17), while an outflow boundary condition was imposed with 50% flow division into the iliac arteries. A rheological model assumed the blood to be an isotropic, incompressible, Newtonian fluid, considered acceptable in the context of large arteries subject to high shear rate, with assigned constant density (1,060 kg/m^3^) and dynamic viscosity (0.00319 Pa·s). The arterial wall was assumed rigid and a no-slip condition was applied at the fluid interface. The convergence criterion was selected after sensitivity analysis and set as 1e^−3^ for the residual of the conserved quantities.

The time-averaged wall shear stress (TAWSS), derived from CFD results, expressed the total amount of shear-stress experienced by the arterial wall throughout the cardiac cycle and was used to quantify local hemodynamic disturbances as described in previous publications (8, 15, 18–20).

### Strain Analysis

*In vivo* three-dimensional principal strain analysis from dynamic CT images was performed using proprietary Matlab-based software, ViTAA (Virtual Touch Aortic Aneurysm–patent WO-2018/068153-A1) to assess aortic wall strength and weakening index. The surface mesh of the outer aortic wall was used as 3D feature-tracking model to measure nodal displacements throughout the cardiac cycle based on an optical flow technique. The deformation gradient, obtained from nodal displacement, allowed for the computation of *in vivo* strain by means of the Green-Lagrange tensor as presented in previous publications (12, 13).

### Regional Aortic Weakness

The RAW index (patent PCT/IB2020/059018) was obtained for the patches defined on each AAA geometry as a combined measure of region-averaged TAWSS obtained from computational fluid dynamic (CFD) simulations, region-averaged ILT thickness, and region-averaged maximum principal strain obtained from the *in vivo* strain analysis. A quartile-based categorization method was applied to the patch-averaged variables of interest and for ease of interpretation a category from 1 to 4 was assigned to each patch for each variable. The category value for the TAWSS was inverted in order to assign a higher risk to low TAWSS regions according to the observations that low TAWSS correlates with ILT deposition, aortic enlargement and rupture (4–6). The RAW index was defined as the sum of the category scores on each single patch in order to capture regions of combined low TAWSS, thick ILT and large strain, and then scaled to obtain a range from 0 to 10 as follow:

$$RAW=\frac{\left[ILT_{category}+STRAIN_{category}+\left(5-\;TAWSS_{category}\right)\right]-3}{9}\cdot 10$$

where *ILT*_*category*_, *STRAIN*_*category*_ and *TAWSS*_*category*_ are the category scores obtained from the quartile-based categorization of the three variables of interest.

### Tissue Samples Collection

Patients selected for open repair underwent aortic resection allowing for the collection of aortic tissue samples through the systematic harvesting of the remaining mid-infrarenal aortic wall after exclusion and replacement with a prosthetic aorta. ILT, if present, was removed prior to sample collection.

Samples intended for uniaxial tensile testing were stored in 1X phosphate-buffered saline (PBS) solution. Samples intended for biaxial tensile testing were marked to indicate orientation and frozen at −80°C. It is worth noticing that previous studies have shown that the mechanical properties of collagen-based tissues are not affected by freezing (21, 22). Samples for fluorescence microscopy analysis were immersed and stored in 4% para-formaldehyde in PBS at 4°C. Samples for histology were immersed and preserved in 10% buffered formalin solution. Samples for microarray analysis were placed into RNAlater solution (Qiagen GmbH, Hilden, Germany) and stored for 24 h at 4°C before long-term storage at −20°C until further RNA isolation.

### Mechanical Testing

The excised aortic samples were analyzed by means of uniaxial tensile test in order to estimate local material properties in the circumferential direction. Uniaxial tests were carried out on a linear motor uniaxial testing system (ElectroForce Systems 3200, TA Instruments, US). Prior to failure, a pre-conditioning cycle of 5 mm/min to 15% strain based on the initial length between grips was performed for 10 cycles at 0.5 Hz. Failure was quantified as the first discontinuity, or maximum, observed on the stress-strain curve. The ultimate tensile strength (UTS) of the tissue was obtained from the load on the specimen at failure.

Biaxial testing was carried out on a four-motor biaxial testing system (ElectroForce Systems, TA Instruments, US), designed to allow independent control of each motor. Full thickness tissue samples were cut into 10 mm × 10 mm squares and mounted to the four linear motors with four suture lines and hooks per side. Using four hooks per side is designed to give an even load distribution along the specimen edge. Five dots were drawn on the central region of the sample using a surgical skin marker to provide distinguishable marks that can be tracked by the overhead camera to obtain the deformation gradient tensor. A high-resolution digital video extensometer (DVE) camera (640 × 480 pixel resolution, 55 mm focal length, 200 frames per second) mounted above the test specimen was used to track the dots, resulting in local measurements. Hook displacement was obtained using motor control, resulting in global measurements. In order to mimic the *in vivo* environment, samples were fully immersed in a PBS solution at 37°C and pH 7.4. A pre-load of 0.05 N was performed to avoid sagging effects on the testing setup and sample loading was recorded using two load cells (22 N). Five different displacement ratios were applied to the specimens at 20, 40, and 60% hook-to-hook displacement. All deformations were obtained from a pre-loaded state and all residual stresses in the tissue were assumed to be released during specimens' excision. A complete and detailed description of the methodology is presented elsewhere (23).

The energy loss for each sample was obtained as a measure of the tissue viscoelasticity following a previously described method by Chung et al. for the ascending aorta (24). The curve of global first Piola-Kirchhoff stress vs. principal stretch was considered for loading and unloading cycles. The energy loss was found as the percentage ratio of the area of hysteresis to the total area under the loading curve.

### Tissue Microscopy and Immunohistochemical Analysis

**Figure 3:**
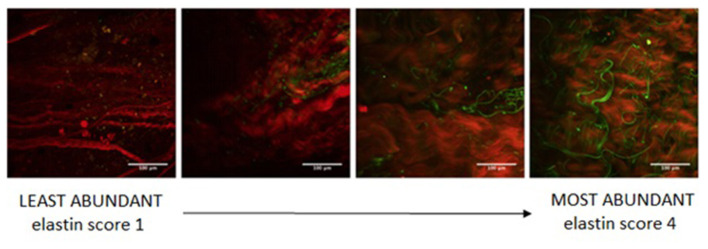
Microscopy samples showing different elastin content (green) to define the elastin abundance scoring system.

Aortic samples were imaged on a multiphoton confocal microscope (Zeiss, Germany) in order to assess morphology and microstructure of the ECM. For capturing second-harmonic generation (SHG), a Ti:Sa chameleon multiphoton tunable laser (Coherent, US) at 780 nm, a custom BP:414/46, DC:495, BP:525/50 filter, a dichroic mirror, and a 20X water immersion objective lens were utilized to capture collagen and elastin in the tissue. Specimens were placed in a petri dish and imaged intima side up. A coverslip was placed over the sample to maintain a flat surface. A minimum depth of 38 μm to a maximum depth of 284 μm was imaged and z-stacks of optical slices at 2 μm spacing were created. Images were processed prior to analysis using FIJI (version 2.0.0-rc-69/1.52n) by splitting the two-photon excitation fluorescence (TPEF) and SHG channels (25). Sample microstructure was evaluated in terms of elastin content, collagen fiber thickness, and collagen fiber directionality by processing signals from TPEF and SHG. Elastin abundance scores were assigned to each z-stack: a value of “1” was assigned to images with little to no visible elastin, while a value of “4” was assigned to images with clear, abundant elastin (Figure 3).

SHG images were then used to quantify collagen fiber directionality using maximum intensity images of the z-stacks.

Immunohistochemical analysis was performed at the Calgary Laboratory Services with a Dako EnVision Flex Detection Kit (Agilent Technologies, US). CD4 (SP35) was run with H15 X 15 at 1/25, CD8 (C8/144B) with H20 X 20, and CD68 (KP1) with H15 X 20. On each whole slide image, an area of the highest stained cell density was identified in order to characterize inflammation in the media and adventitia layers. An area of 1 mm^2^ was selected, and positively stained cells were manually counted for each marker: helper T-cells (CD4+), cytotoxic T-cells (CD8+), and macrophages (CD68+).

### RNA Analysis

Tissues samples collected for transcriptomic analysis were suspended in MirVana Lysis/Binding buffer (ThermoFisher, US), and homogenized using a rotor-type homogenizer (Tissue-Tearor, Biospec Products, US) for 6 min. Samples were then centrifuged at 500*g* for 2 min, and finally homogenized for another 6 min. After homogenization, RNA was purified from the samples using MirVana miRNA isolation kit. Isolated RNA was stored at −80°C and then analyzed using an Affymetrix Human Transcriptome Array (HTA 2.0) (ThermoFisher, US), or a Clariom D microarray (ThermoFisher, US). The files generated from the microarray experiments were analyzed using Transcriptome Analysis Console (TAC) software developed by Affymetrix (ThermoFisher, US), as well as the Ingenuity Pathway Analysis software package (Qiagen GmbH, Germany). Raw microarray data was uploaded to the Genevestigator database. The quality and integrity of RNA samples were assessed by an Agilent Bioanalyzer 2100 prior to microarray analysis.

### Statistical Analysis

Statistical analysis was performed in MATLAB (MathWorks, US) and included: Shapiro-Wilk test for normality, ANOVA (or corresponding Kruskal-Wallis test for non-normal distribution after failed transformation) to test the influence of patch position and patient on the variable of interest, unpaired *t*-test (or corresponding non-parametric Mann-Whitney *U* test) to evaluate the effect of patch orientation (i.e., left/right and anterior/posterior) on the different variables. Parametric Pearson's correlation or non-parametric Spearman's rank correlation were employed to examine the strength (and direction) of, respectively, linear or simply monotonic relationships between variables. Statistical significance was tested for *p*-values < 0.05 (*p*-value reported for *t*-test or non-parametric Mann-Whitney *U* test as two-tailed).

As further validation, the performance of the proposed RAW index as a classifier of aortic risk was assessed by means of area under the receiver operating characteristics curve (AUC).

A *p*-value cut-off of 0.05, a fold change of two, and a gene level false discovery rate (FDR) value of 0.05 were used for gene expression analysis.

## Results

Between January 2016 and February 2019, 23 AAA patients selected for aortic repair consented to participate in the study. One patient was excluded from the analysis due to the poor quality of the dynamic CT images for *in vivo* strain analysis.

The AAA study population (*n* = 22, age 69 ± 7 years, 86% males) had a maximum aortic diameter of 53.4 ± 9.0 mm. Five patients (23%) presented a maximum diameter under the threshold for elective intervention (50 mm for women and 55 mm for men), however they showed complications such as penetrating aortic ulcer or acute ischemic leg and were therefore considered critical and deemed to receive aortic repair. Ten patients in the population received open repair surgery and 12 were treated with EVAR. Mean length of stay for open aortic patients was 7.7 days and was unchanged compared to non-resected open aortic surgery cohorts. In addition, blood loss for the aortic resection patients was minimal due to the aortic sac not being opened during the procedure. One post-operative death due to a post-operative myocardial infarction was unrelated to the aortic reconstruction. There were no significant complications observed in the endovascular repair group.

### *In Vivo* Analysis: Region-Averaged Descriptors and Raw Index

**Figure 4:**
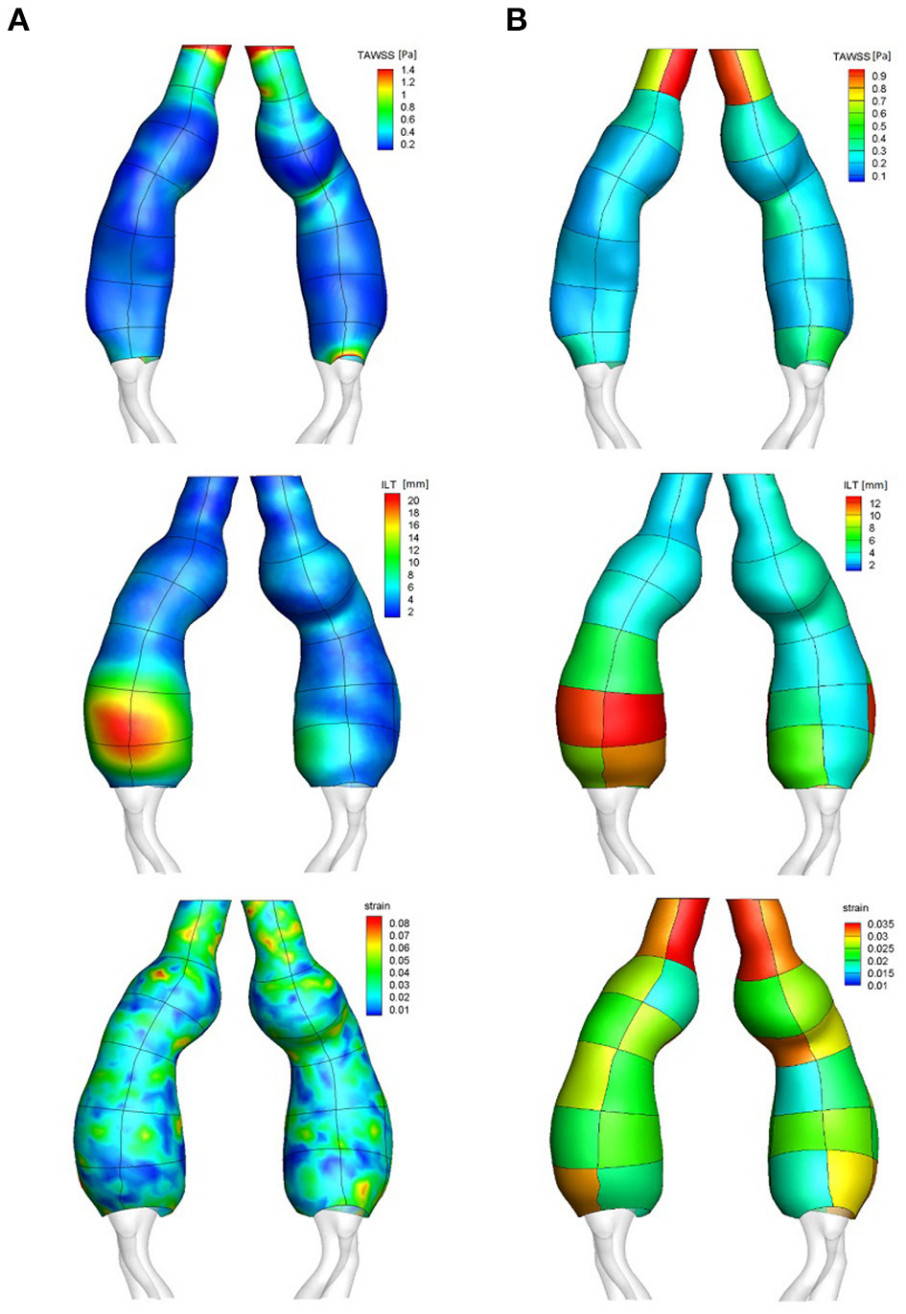
Surface distribution (column **A**) with patches outline visible on top of the distribution, and patch-averaged distribution (column **B**) of TAWSS, ILT thickness and maximum principal strain: case example.

Figure 4 shows an AAA case example with the CFD-derived luminal distribution of TAWSS, outer wall distribution of maximum principal strain and ILT thickness, and the obtained region-averaged distribution for the same parameters.

The mean TAWSS for the population was found to be 0.52 ± 0.25 Pa with a significant interpatient variability (*p* < 0.001). The patch position along the vessel also had a significant effect on the TAWSS (*p* < 0.001) as central patches exhibited lower TAWSS reflecting low velocity and recirculation patterns in the aneurysmal sac, in contrast to the faster blood flow upstream and downstream the enlargement. The patch orientation (right/left and anterior/posterior) was found to have no significant effect on the TAWSS regional distribution; of note a trend toward lower TAWSS was observed for anterior patches (0.50 ± 0.24 Pa for anterior orientation vs. 0.54 ± 0.25 Pa for posterior orientation, *p* = 0.054).

The mean ILT thickness for the population was 4.6 ± 3.8 mm. Significant interpatient variability was observed (*p* < 0.01) along with an effect of position on the ILT thickness, with an increased ILT thickness observed at central patches in the aneurysms (*p* < 0.01).

Finally, the maximum principal strain (population mean 0.03 ± 0.01) also presented significant interpatient variability as well as an influence of position (both *p* < 0.001), with central patches located in dilated region of the vessel showing smaller strain.

A positive correlation was found between maximum aortic diameter and maximum ILT thickness (Pearson's *r* = 0.64, *p* = 0.001).

The predicted flow patterns were generally characterized by recirculation and low velocities at the aneurysmal sac of individual aortas, where low TAWSS values and thick ILT predominated: a negative correlation was found between region-averaged TAWSS and ILT thickness (Spearman's ρ = −0.34, *p* < 0.001). A main flow channel associated with high velocity was commonly found in the neck and in areas of flow impingement on the aortic wall where it resulted in high TAWSS, almost no ILT and high strain, pointing to the correlation found between region-averaged TAWSS and maximum principal strain (Spearman's ρ = 0.24, *p* < 0.001), and between region-averaged ILT and strain (Spearman's ρ = −0.36, *p* < 0.001).

**Figure 5:**
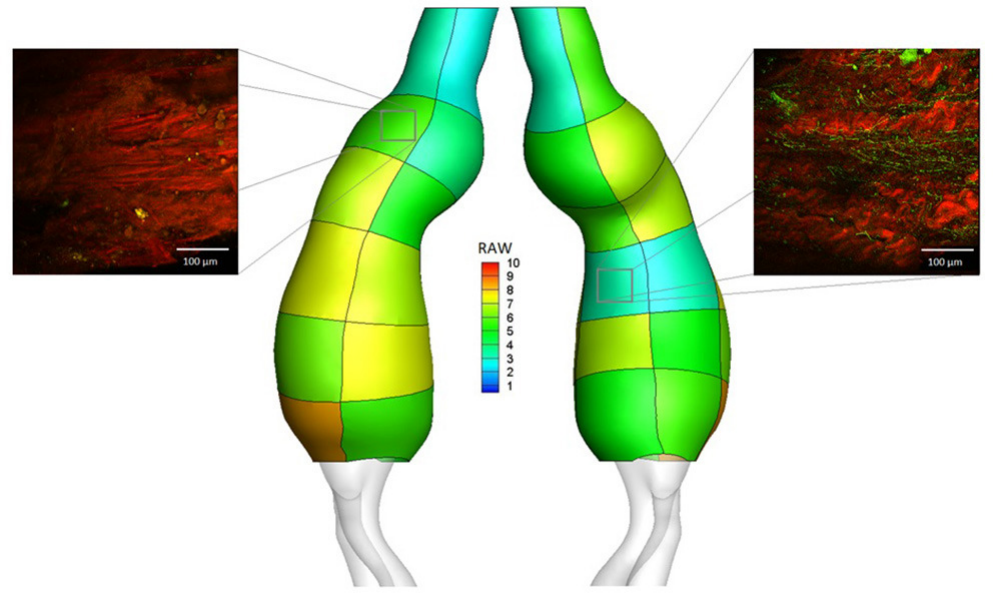
RAW distribution on regions defined on the external wall for the AAA case example. Micrographs are reported for two of the collected samples: region LA2 (RAW = 5.56, elastin abundance score 1, Ultimate Tensile Strength from uniaxial tensile test UTS = 0.21 MPa) and region RP4 (RAW = 3.33, elastin abundance score 4, Ultimate Tensile Strength from uniaxial tensile test UTS = 0.86 MPa). Microscopy analysis was performed on a total of 73 aortic samples; uniaxial tensile testing was performed on a total of 97 aortic samples.

**Figure 6:**
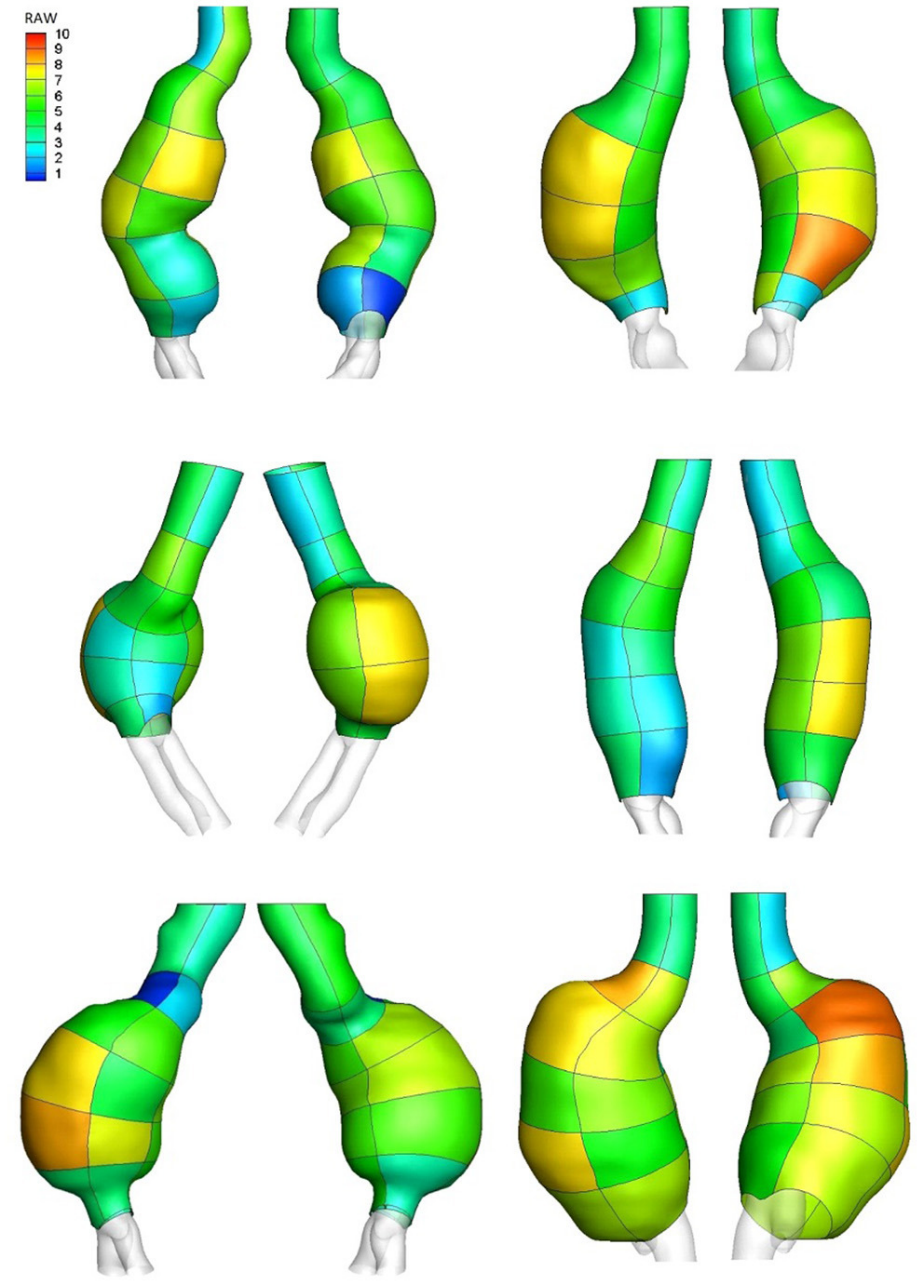
RAW distribution on regions defined on the external wall of AAA case examples.

Figure 5 shows the RAW index distribution obtained on the external wall of the same AAA case example; additional RAW distributions for AAAs in the study population can be found in Figure 6. A region characterized by low TAWSS, thick ILT, and large maximum principal strain corresponds to a high RAW index indicating a weak wall.

### *Ex Vivo* Analysis

Ten patients in the study population were deemed to receive open repair surgery involving complete aortic resection. One of these patients showed early signs of impending aortic rupture during pre-operative imaging and underwent emergency open repair surgery. Sample collection was not feasible for this subject; however, the proposed combined analysis was performed and the novel index was found to identify the impending rupture location. This case report is presented in a separate publication (15). Aortic samples were therefore collected for nine patients (*n* = 9, age 65 ± 7 years, average maximum aortic diameter 4.8 ± 0.9 cm). Table 1 reports patients' details for the AAA open repair subgroup.

**Table 1:** Patients' details for the AAA open repair population with collected aortic samples.

**Patient**	**History of smoking**	**Hypertension**	**Co-morbidities**	**AAA diameter[cm]**	**Age [years]**	**Gender**
1	no	yes	penetrating atherosclerotic ulcer × 2	4.2	52	m
2	yes	no	penetrating atherosclerotic ulcer	5.0	60	m
3	yes	yes	–	3.9	65	m
4	yes	no	acute left ischemic leg, chronic thrombus	3.2	62	m
5	no	yes	–	5.4	67	m
6	yes	yes	peripheral artery disease	4.4	68	f
7	yes	yes	dyslipidemia, coronary artery disease, peripheral artery disease	5.3	75	m
8	yes	no	dyslipidemia	5.5	64	m
9	yes	yes	dyslipidemia	6.2	72	m

### Mechanical Testing and Raw Index

Uniaxial tensile test was performed on 97 aortic tissue samples. The mean UTS for the analyzed samples was 0.71 ± 1.06 MPa. Samples were classified into two groups based on strength estimate, with a UTS equal to 0.3 MPa being set as threshold to define low regional strength. The threshold choice was based on previously reported values obtained from testing on *ex vivo* samples of ruptured AAAs (26). The RAW index was significantly higher for patches exhibiting low UTS estimated invasively (5.89 ± 1.95 vs. 5.04 ± 1.82 for low and high UTS, respectively; *p* = 0.035). The RAW index had an AUC for the constructed receiver operating characteristic curve of 0.61 (95% confidence interval: 0.48, 0.73, *p* = 0.043).

Thirty-five samples were collected for biaxial tensile testing, of which 13 were excluded due to rupture during testing set-up or initial testing protocol, and two were discarded post-analysis due to post-processing failure. Thus, biaxial tensile test was performed on a total of 20 aortic samples. The mean energy loss for the specimens was 34.86 ± 3.99% in the longitudinal direction and 34.07 ± 3.73% in the circumferential direction. Samples characterized by a high RAW index indicating regional weakening (RAW > 6 according to UTS values) exhibited higher energy loss in both directions, with statistically significant differences observed in the longitudinal direction (37.66 ± 4.64% vs. 33.51 ± 3.14% for RAW > 6 and RAW < 6 respectively; *p* < 0.040).

### Tissue Microscopy and Raw Index

Microscopy analysis was performed on 73 collected aortic samples. The mean elastin abundance score for the analyzed samples was 2.4 ± 1.2. Samples were classified into two groups based on elastin content, with score values ≥ 3 indicating high elastin abundance and score values < 3 indicating low elastin abundance. Samples characterized by a low elastin score presented significantly higher RAW index (5.85 ± 1.76 vs. 4.88 ± 1.66 for low and high elastin abundance, respectively; *p* = 0.020). Figure 5 shows the microscopy and elastin abundance of two tissue samples for the AAA case example and the corresponding RAW index.

The mean collagen fibers directionality and thickness for the analyzed samples were −2 ± 21° and 6.7 ± 3.9 μm, respectively. No significant RAW difference was found when samples were classified according to the microstructural properties of collagen fibers.

### Inflammation and Raw Index

Of the initial 73 aortic samples collected for microscopy and immunohistochemical analysis, a subset of 38 samples were used for cell count, while the remaining samples had to be excluded due to absent or extremely thin media layer making the analysis not feasible. Significantly higher content of inflammatory infiltrate was present in the adventitia compared to the media layers of the aortic samples for all three cell types (CD4+ *p* < 0.05; CD8+ *p* < 0.05; CD68+ *p* < 0.01).

For each cell type, the median value of cell counts was used to classify samples based on high and low inflammatory content in both the media and adventitia layers. The RAW index was generally higher for samples presenting high inflammatory marker content; this trend, however, did not reach statistical significance.

No significant correlation was observed between inflammatory cell count values and ILT thickness.

### Gene Expression in Constitutive Metrics

A subset of aortic samples (*n* = 10) were determined to be of sufficient quality for RNA analysis, with a mean RNA integrity number (RIN) of 5 ± 1.2. Table 2 reports a summary of the patch-averaged descriptors, assigned quartiles and the final RAW values for the samples analyzed for gene expression.

**Table 2:** Summary of RNA integrity values, raw patch-averaged descriptors, assigned quartiles, as well as final RAW values for samples used in gene expression analysis.

**Patient**	**Sample**	**RIN**	**patch-averaged DATA**			**Combined quartile**			**RAW**
			**Strain**	**ILT**	**TAWSS**	**Strain**	**ILT**	**TAWSS**	
1	LA5	4.2	0.085	2.58	0.15	4	2.5	1	8.33
4	LA1	7.6	0.013	1.99	0.64	1	1	3	1.11
	RA1	5.4	0.027	2.32	0.59	2.5	1	3	2.78
	RA2	4.9	0.027	2.14	0.58	2.5	1	3	2.78
5	LP1	4.6	0.040	3.89	0.44	4	2.5	2.5	6.67
9	LA3	5.3	0.018	2.90	0.54	1	1.5	3	1.67
	LA6	4.2	0.023	21.22	0.31	2.5	4	1.5	7.78
	RP4	4	0.021	12.79	0.30	1.5	4	1.5	6.67
	RA5	4.5	0.020	12.68	0.32	1.5	4	1.5	6.67
	RP6	6.7	0.024	5.84	0.30	2.5	3	1.5	6.67

**Figure 7:**
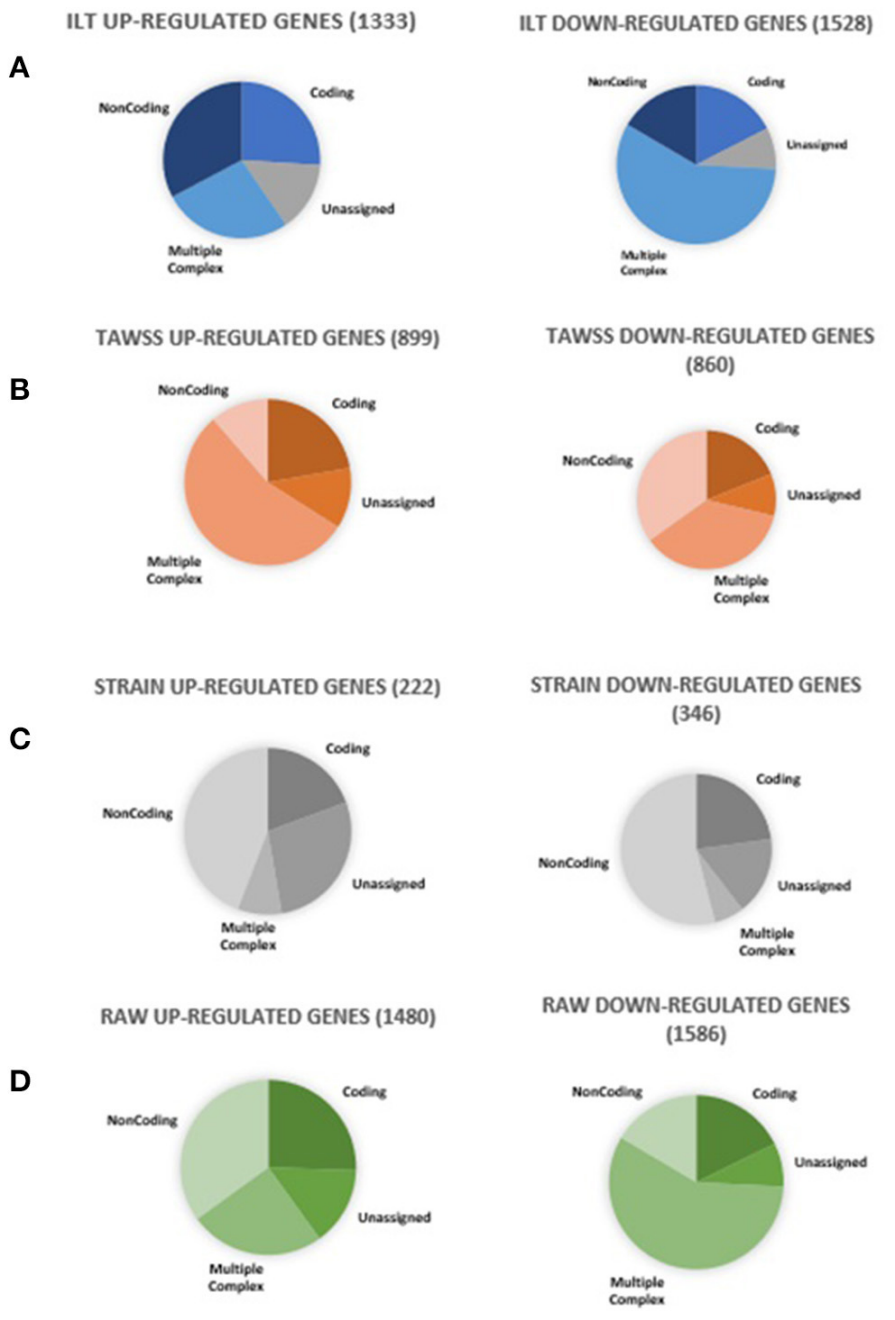
Summary of genes differentially expressed in RAW and all its constitutive metrics. Ten aortic samples were analyzed, with one microarray being performed on each tissue sample.

Gene expression as a function of each metric (ILT, TAWSS, and strain) was examined by analysis of genes differentially expressed and the gene ontology pathways affected. ILT thickness was found to have a large effect on gene expression, with 2,861 genes differentially expressed (Figure 7); these genes clustered based on ILT. Samples were classified as either having a high metric (combined quartile accounting for both patient-specific and population-based distribution > 2) or a low metric (combined quartile < 2). TAWSS analysis showed 1,759 genes differentially expressed, however the genes did not cluster based on TAWSS. With strain, only 568 genes were differentially expressed, but did cluster based on strain.

Gene ontology analysis (Table 3) for the ILT metric showed significant changes in genes involved in cell survival, movement and death as well as cytoskeleton reorganization, which are all involved in AAA development and progression. For the TAWSS, genes involved in leukocyte migration, cellular immune response, lymphocytes, macrophages and cytotoxicity dominated in the analysis (Table 4). Despite having 568 differentially expressed genes, the strain did not show any gene ontology pathways being significantly altered, as well as no changes in genes previously found to be relevant to AAA.

**Table 3:** Gene ontology pathways affected by thick ILT deposition. Ten aortic samples were analyzed, with one microarray being performed on each tissue sample.

**Expressed pathway**	**Activation Z-score**	**Genes altered**
Cell survival	−5.504	132 of 208 genes have a measurement direction consistent with a **decrease** in cell survival in high ILT samples
Cell movement	−5.353	215 of 419 genes have a measurement direction consistent with a **decrease** in cell movement in high ILT samples
Reorganization of cytoskeleton	−3.575	23 of 43 genes have a measurement direction consistent with a **decrease** in reorganization of cytoskeleton in high ILT samples
Apoptosis	2.391	180 of 378 genes have a measurement direction consistent with an **increase** in apoptosis in high ILT samples
Cell death of muscle cells	2.679	35 of 57 genes have a measurement direction consistent with an **increase** in the cell death of muscle cells in high ILT samples

**Table 4:** Gene expression pathways affected by low TAWSS. Ten aortic samples were analyzed, with one microarray being performed on each tissue sample.

**Expressed pathway**	**Activation Z-score**	**Genes altered**
Leukocyte migration	−5.5087	79 of 151 genes have a measurement direction consistent with a **decrease** in leukocyte migration in low TAWSS samples
Immune response of cells	−4.226	51 of 77 genes have a measurement direction consistent with a **decrease** in immune response of cells in low TAWSS samples
Quantity of CD4+ T-lymphocytes	−3.940	31 of 40 genes have a measurement direction consistent with a **decrease** in quantity of CD4+ T-lymphocytes in low TAWSS samples
Cytotoxicity of cells	−3.680	24 of 32 genes have a measurement direction consistent with a **decrease** in cytotoxicity of cells in low TAWSS samples
Immune response of macrophages	−2.616	16 of 24 genes have a measurement direction consistent with a **decrease** in immune response of macrophages in low TAWSS samples

### Gene Expression and Raw Index

Samples were classified based on RAW index by using a value of 6 as threshold to define low and high level of weakening based on the mean RAW (5.89) found for samples characterized by low UTS (<0.3 MPa). The RAW index had the greatest number of genes differentially expressed compared to any individual metric (i.e., TAWSS, ILT, strain). Out of 3,066 genes differentially expressed, 1,480 were up-regulated and 1,586 down-regulated. A *p*-value cut-off of 0.05 was used, along with a fold change cut-off of two.

**Figure 8:**
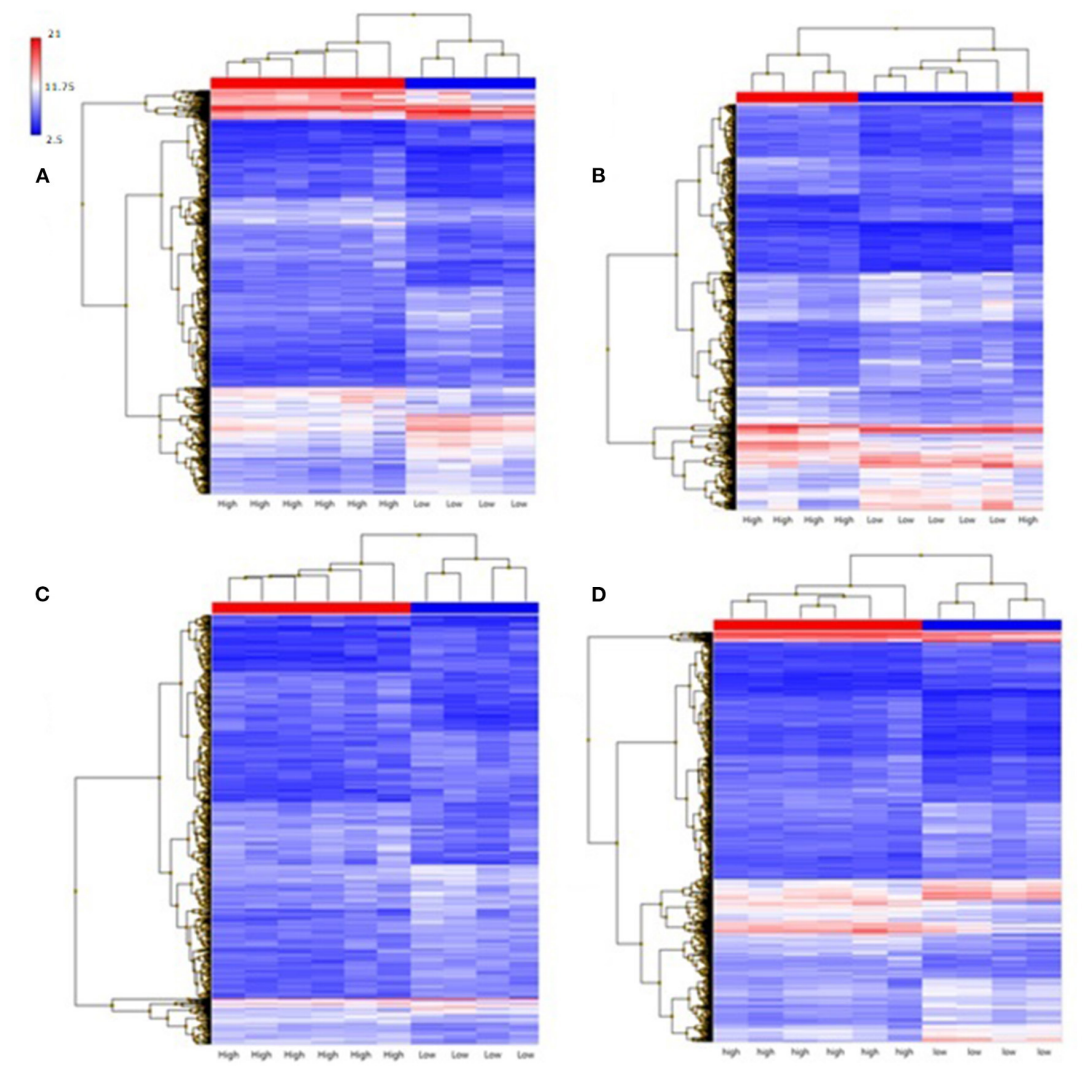
Hierarchical clustering of gene expression of aneurysmal tissues, samples classified as high are red and samples classified as low are blue. **(A)** ILT; **(B)** TAWSS; **(C)** Strain; **(D)** RAW. 10 aortic samples were analyzed, with one microarray being performed on each tissue sample.

The primary gene ontology pathways affected by a high RAW (Table 5) are those regulating cell movement of smooth muscle and endothelial cells (down-regulated), reorganization of cytoskeleton (down-regulated), and finally, angiogenesis (down-regulated), suggesting that there are microstructural changes occurring in high risk AAA samples. A selection of genes differentially expressed by high RAW samples and known to be relevant to AAA are presented in Table 6; heat maps showing gene expression for RAW, as well as all constitutive metrics, are shown in Figure 8.

**Table 5:** Gene ontology pathways affected by high RAW. Ten aortic samples were analyzed, with one microarray being performed on each tissue sample.

**Expressed pathway**	**Activation Z-score**	**Genes altered**
Cell movement	−5.668	218 of 417 genes have a measurement direction consistent with a **decrease** in Cell Movement in high RAW samples.
Cell movement of endothelial cells	−5.361	62 of 85 genes have a measurement direction consistent with a **decrease** in endothelial cell movement in high RAW samples
Cell movement of smooth muscle cells	−3.202	25 of 39 genes have a measurement direction consistent with a **decrease** in smooth muscle cell movement in high RAW samples
Reorganization of cytoskeleton	−3.462	22 of 42 genes have a measurement direction consistent with a **decrease** in Reorganization of Cytoskeleton in high RAW samples
Angiogenesis	−3.807	94 of 190 genes have a measurement direction consistent with a **decrease** in angiogenesis in high RAW samples

**Table 6:** Selection of genes relevant to AAA differentially expressed by high RAW samples. Ten aortic samples were analyzed, with one microarray being performed on each tissue sample.

**Gene symbol**	**Fold change**	**Description**	**Associations**
FLNA	−15.53	filamin A, alpha	Cardiovascular disease
TGFB2, TGFB2-OT1	−6.7	transforming growth factor beta 2; TGFB2 overlapping transcript 1	Cytokine
PDK2	−5.16	pyruvate dehydrogenase kinase, isozyme 2	Immune response
PDK3	−4.76	pyruvate dehydrogenase kinase, isozyme 3	Immune response
ILK	−3.93	integrin linked kinase	Cytokine
MMP16	−3.62	matrix metallopeptidase 16 (membrane-inserted)	Matrix remodeling
TGFB1I1	−3.53	transforming growth factor beta 1 induced transcript 1	Cytokine
TIMP4	−3.25	TIMP metallopeptidase inhibitor 4	Matrix remodeling
TGFB2-AS1	−2.68	TGFB2 antisense RNA 1 (head to head)	Cytokine
PLAT	−2.67	plasminogen activator, tissue	Matrix remodeling
ELN	−2.5	elastin	Matrix remodeling
S100A11	−2.27	S100 calcium binding protein A11	Cytokine
S100A7A	−2.01	S100 calcium binding protein A7A	Cytokine
COL24A1	2.32	collagen, type XXIV, alpha 1	Matrix remodeling
COL10A1	2.33	collagen, type X, alpha 1	Matrix remodeling
CASP4	3.06	caspase 4	Apoptosis
CASP1	3.29	caspase 1	Apoptosis
PDK4	3.6	pyruvate dehydrogenase kinase, isozyme 4	Immune response
S100A9	4.09	S100 calcium binding protein A9	Cytokine
PDK1	4.36	pyruvate dehydrogenase kinase, isozyme 1	Immune response
C3	4.83	complement component 3	Immune response
S100A8	5.44	S100 calcium binding protein A8	Cytokine
MMP12	13.04	matrix metallopeptidase 12	Matrix remodeling
IGHA1	15.39	immunoglobulin heavy constant alpha 1	Immune response
COL11A1	15.8	collagen, type XI, alpha 1	Matrix remodeling
COL6A3	18.23	collagen, type VI, alpha 3	Matrix remodeling
IGHA2	75.21	immunoglobulin heavy constant alpha 2 (A2m marker)	Immune response

## Discussion

This study aimed at correlating the local aortic microstructure with the local aortic mechanics and gene expression. A non-invasive surrogate measure of regional aortic weakening, RAW, was used to assess a population of AAA patients. Non-invasive regional data were correlated with results from uniaxial and biaxial tensile tests, transcriptomic and microscopy analysis performed on corresponding *ex vivo* aortic samples systematically collected to allow the tracking of their location.

Low TAWSS, thick ILT, and large strain were found to play a role in defining regional weakening. The RAW index was able to capture biomechanical changes linked to the weakening of the aortic wall, as assessed through mechanical tests and confirmed by the decrease in elastin content found by microscopy. The index was also able to expose structural changes, such as decrease in gene expression of elastin. High-risk samples, identified by a high RAW index, exhibited significant changes in gene expression related to disease progression and showed the greatest number of genes differentially expressed compared to any individual metric.

From the *in vivo* analysis, a positive correlation between maximum aortic diameter and maximum thrombus thickness suggests a mechanism of ILT accumulation to compensate for vessel expansion, in agreement with previous literature findings (4, 27). This mechanism appears to be associated with areas of disturbed blood flow, characterized by low TAWSS as demonstrated by the negative correlation between patch-averaged ILT thickness and TAWSS. Although the shear stress is unlikely to cause aneurysm rupture, it may contribute to ILT accumulation, which, in turn, may promote local inflammatory processes and hypoxia leading to the adverse remodeling and loss of structural integrity that drives disease progression (28–30). Additionally, the ILT was responsible for the largest change in gene expression among individual metrics that were studied.

Heterogeneous remodeling was reflected by local *in vivo* deformation as a result of heterogeneous material properties. The positive regional correlation between TAWSS and strain may be a consequence of flow impingement on the wall, especially in cases where the lumen suddenly expands away from the neck. Similarly, the negative correlation between region-averaged ILT thickness and maximum principal strain suggests a reinforcement effect of the thrombus. These correlations point at areas of large strain behind thick thrombus as indicative of regional weakening in the aortic tissue.

From *ex vivo* analysis, the RAW index (defined to capture areas of low TAWSS, thick ILT and large strain) was able to differentiate weaker wall regions that exhibited low UTS estimated invasively through uniaxial tensile testing. Regions characterized by high RAW (>6) exhibited higher energy loss derived from biaxial tensile testing, suggesting a more viscoelastic behavior of the tissue. Energy loss has been reported to increase with aneurysm size and to strongly correlate with histological changes in thoracic aortic aneurysms, such as advanced medial degeneration, and greater collagen-to-elastin ratios (24). While it is unclear whether greater energy loss promotes wall remodeling or if it occurs as a consequence of remodeling, present results point at an association between increased energy loss and regional aortic weakening.

High RAW index samples showed a decrease in gene expression for elastin, as well as an increase in expression for matrix metalloproteinase-12 (MMP12) which degrades elastin in the ECM (16, 31, 32). This is further compounded by an increase in expression for tissue inhibitor of metalloproteases-4 (TIMP4), suggesting heterogeneous matrix remodeling throughout the aorta, with high RAW index samples undergoing elastin degradation without replacement. This finding is reflected in the significantly higher RAW index in tissue samples showing low elastin abundance score. Previous investigations have characterized this method of AAA progression: elastin content has been found to be lower in aneurysms compared to healthy tissue, and ruptured AAAs found to have an even lower elastin content than non-ruptured ones (33–36). However, elastin content specifically at the site of rupture compared to non-ruptured sections of AAA has not been studied. The degradation and decrease in elastin suggest that trends occurring at entire-aneurysm scale are potentially repeated locally throughout the aneurysm, leading to focal areas of local weakness throughout the aorta.

Other studies have suggested the underlying cause of AAA progression and eventual destabilization to be local inflammation and infiltration by immune cells (1). However, high RAW index samples did not show a significant increase in either gene expression pathways leading to inflammation and immune responses, or a significant increase in immune cell infiltration. While AAA has been shown to be pro-inflammatory, there is some evidence that inflammation may be a non-specific process and there may be very little difference in regional inflammation throughout the aneurysm (37). An alternative trigger to the elastin degradation that leads to AAA destabilization may be hypoxia: as ILT thickens and the perfusion distance for oxygen and other nutrients to reach the aortic wall from the lumen increases, endothelial cells experience hypoxia, which can lead to apoptosis and cell death (28–30). Samples with a high RAW index showed gene expression patterns related to angiogenesis and an increase in cell death and apoptosis. Samples not undergoing angiogenesis to compensate for decreased luminal perfusion may be at higher risk of hypoxia and therefore cell death. This suggests that high risk samples may be characterized by a decrease in *vasa vasorum* thickness, rather than the typical increase generally reported by the literature.

The presented methodology is not the first suggesting a biomechanics-based index aimed at improving prediction of AAAs risk of growth and rupture; (10, 11) however, this is the first study proposing an index that did not introduce modeling assumption on aortic and ILT material properties, and reached statistical significance while capturing changes in the tissue mechanical behavior, microstructure and gene expression. It is important to note how this study's unique methodology, involving surgery with complete aortic resection, allowed the collection of a number of aortic specimens from different regions of each individual aneurysm, therefore accounting for the regional variability and tissue heterogeneity, despite the small AAA population. While previous studies have investigated histological and mechanical changes in aneurysmal tissue, these analyses were performed on a single specimen assumed to be representative of the entire aneurysm.

AAA surgical repair is usually performed at a late stage of aneurysm progression, when the risk of rupture surpasses the risk of surgery related complications. Consequently, *ex vivo* studies mainly focus on late stage AAAs, therefore overlooking important information that may be present at early stages of tissue remodeling. While a limitation of this study is that tissue samples were only obtained from patients not suitable for endovascular procedure due to their geometric complexity or other factors, no significant differences were found between the open and endovascular repair groups in terms of calculated RAW index (*p* = 0.353).

There are some limitations to the study, such as the assumption of rigid aortic wall in CFD simulations and the use of uniaxial testing to characterize the tissue mechanical properties, but we believe that the novel aspects and outcomes of the present investigation compensate for these concerns. Moreover, the assumption of a rigid aortic wall for CFD simulations is accurate in capturing the main flow features when considering a highly heterogeneous structure (aortic wall and ILT) with unknown patient-specific material properties and the increased computational cost linked to simulations with moving walls (38). While a uniaxial test cannot fully characterize the mechanical properties of the aortic tissue due to anisotropy and presence of fibers, tissue strength can be evaluated from the maximum stress at failure and was used in this study as a surrogate of propensity for rupture (7, 26, 39).

Though elastin abundance was assessed for each sample, it would be valuable to conduct further study into elastin fragmentation in addition to elastin abundance as well as collagen to help better discriminate the individual specimen remodeling stage. Moreover, the assessment of elastin content can be improved in order to introduce a more automatic and objective approach compared to the visual one.

Finally, the current investigation is limited to a small cohort of AAA patients; as future work, the described approach will be extended to a larger population to better account for the large inter-patient variability. Future work will also aim at introducing predictive models and incorporate a machine learning based approach in order to improve the present non-invasive surrogate measure of regional aortic weakening.

Present findings are the result of a unique study that enables acquisition of tissue from multiple regions in the aortic aneurysm to assess the role of tissue properties on transcriptomic profiles, and show the importance of regional descriptors when assessing aortic wall vulnerability in the context of aneurysm monitoring. The proposed approach provides a meaningful insight on aortic weakening that may assist in estimating the risk of growth and rupture for individual aneurysms. Implementation of new approaches that can better identify which individuals have higher risks of rupture can improve disease management and patient outcomes.

## Data Availability Statement

The original microarray data presented in the study is publicly available and can be found on the GEO repository: https://www.ncbi.nlm.nih.gov/geo/query/acc.cgi?acc=GSE165470.

## Ethics Statement

The studies involving human participants were reviewed and approved by the University of Calgary Conjoint Health Research Ethics Board (CHREB - Ethics ID #REB15-0777). The patients/participants provided their written informed consent to participate in this study. Written informed consent was obtained from the individual(s) for the publication of any potentially identifiable images or data included in this article.

## Author Contributions

ED and KR: study conception and design. RM: patients enrollement. AF, JK, ED, AI, RS, DS, and RM: tissue samples collection. AI: *ex vivo* mechanical testing. JK, RS, and DS: *ex vivo* RNA analysis. AI and AB: *ex vivo* microscopy and immunohistochemical analysis. AF: *in vivo* analysis (including CFD, strain, ILT). AF, JK, ED, and KR: statistical analysis and interpretation. AF and JK: manuscript writing and preparation. AF, JK, AI, RS, DS, AB, RM, ED, and KR: manuscript critical revision and approval. All authors contributed to the article and approved the submitted version.

## Conflict of Interest

ED, RM, and AF are cofounders and shareholders of the start-up company ViTAA. The remaining authors declare that the research was conducted in the absence of any commercial or financial relationships that could be construed as a potential conflict of interest.

## References

[1] Kuivaniemi H Ryer EJ Elmore JR Tromp G. Understanding the pathogenesis of abdominal aortic aneurysms. Expert Rev Cardiovasc Ther. (2015) 13:975–87. 10.1586/14779072.2015.1074861 26308600 PMC4829576

[2] Brown PM Pattenden R Vernooy C Zelt DT Gutelius JR. Selective management of abdominal aortic aneurysms in a prospective measurement program. J Vasc Surg. (1996) 23:213–22. 10.1016/S0741-5214(96)70265-3 8637098

[3] Brown LC Powell JT. Risk factors for aneurysm rupture in patients kept under ultrasound surveillance. Ann Surg. (1999) 230:289–97. 10.1097/00000658-199909000-00002 10493476 PMC1420874

[4] Zambrano B Gharahi H Lim C Jaberi F Choi J Lee W. Association of intraluminal thrombus, hemodynamic forces, and abdominal aortic aneurysm expansion using longitudinal CT images. Ann Biomed Eng. (2016) 44:1502–14. 10.1007/s10439-015-1461-x 26429788 PMC4826625

[5] Boyd AJ Kuhn DCS Lozowy RJ Kulbisky GP. Low wall shear stress predominates at sites of abdominal aortic aneurysm rupture. J Vasc Surg. (2016) 63:1613–9. 10.1016/j.jvs.2015.01.040 25752691

[6] Meyrignac O Bal L Zadro C Vavasseur A Sewonu A Gaudry M. Combining volumetric and wall shear stress analysis from CT to assess risk of abdominal aortic aneurysm progression. Radiology. (2020) 295:722–9. 10.1148/radiol.2020192112 32228297

[7] Vallabhaneni SR Gilling-Smith GL How TV Carter SD Brennan JA Harris PL. Heterogeneity of tensile strength and matrix metalloproteinase activity in the wall of abdominal aortic aneurysms. J Endovasc Ther. (2004) 11:494–502. 10.1583/04-1239.1 15298501

[8] Martufi G Forneris A Nobakht S Rinker KD Moore RD Di Martino ES. Case study: intra-patient heterogeneity of aneurysmal tissue properties. Front Cardiovasc Med. (2018) 5:82. 10.3389/fcvm.2018.00082 30018968 PMC6037694

[9] Di Martino ES Guadagni G Fumero A Ballerini G Spirito R Biglioli P. Fluid-structure interaction within realistic three-dimensional models of the aneurysmatic aorta as a guidance to assess the risk of rupture of the aneurysm. Med Eng Phys. (2001) 23:647–55. 10.1016/S1350-4533(01)00093-5 11755809

[10] Vande Geest JP Di Martino ES Bohra A Makaroun MS Vorp DA. A biomechanics-based rupture potential index for abdominal aortic aneurysm risk assessment: demonstrative application. Ann N Y Acad Sci. (2006) 1085:11–21. 10.1196/annals.1383.046 17182918

[11] Stevens RRF Grytsan A Biasetti J Roy J Liljeqvist ML Christian Gasser T. Biomechanical changes during abdominal aortic aneurysm growth. PLoS ONE. (2017) 12:e0187421. 10.1371/journal.pone.0187421 29112945 PMC5675455

[12] Satriano A Rivolo S Martufi G Finol EA Di Martino ES. *In vivo* strain assessment of the abdominal aortic aneurysm. J Biomech. (2015) 48:354–60. 10.1016/j.jbiomech.2014.11.016 25497379

[13] Satriano A Guenther Z White J Merchant N Di Martino ES Al-Qoofi F. Three-dimensional thoracic aorta principal strain analysis from routine ECG-gated computerized tomography: feasibility in patients undergoing transcatheter aortic valve replacement. BMC Cardiovasc Disord. (2018) 18:76. 10.1186/s12872-018-0818-0 29720088 PMC5932860

[14] Pasta S Agnese V Di Giuseppe M Gentile G Raffa GM Bellavia D. *In vivo* strain analysis of dilated ascending thoracic aorta by ECG-gated CT angiographic imaging. Ann Biomed Eng. (2017) 45:2911–20. 10.1007/s10439-017-1915-4 28884233

[15] Forneris A Marotti FB Satriano A Moore RD Di Martino ES. A novel combined fluid dynamic and strain analysis approach identified abdominal aortic aneurysm rupture. J Vasc Surg Cases Innov Tech. (2020) 6:172–6. 10.1016/j.jvscit.2020.01.014 32322769 PMC7160522

[16] Erhart P Schiele S Ginsbach P Grond-Ginsbach C Hakimi M Böckler D. Gene expression profiling in abdominal aortic aneurysms after finite element rupture risk assessment. J Endovasc Ther. (2017) 24:861–9. 10.1177/1526602817729165 28856923

[17] Mills C Gabe I Gault J Mason D Ross J Braunwald E. Pressure-flow relationships and vascular impedance in man. Cardiovasc Res. (1970) 4:405–17. 10.1093/cvr/4.4.405 5533085

[18] Gallo D Isu G Massai D Pennella F Deriu MA Ponzini R. A survey of quantitative descriptors of arterial flows. In: Lima R Imai Y Ishikawa T Oliveira M editors. Visualization and Simulation of Complex Flows in Biomedical Engineering. Lecture Notes in Computational Vision and Biomechanics. Dordrecht: Springer (2014). p. 1–24. 10.1007/978-94-007-7769-9_1

[19] Pasta S Agnese V Gallo A Cosentino F Di Giuseppe M Gentile G. Shear stress and aortic strain associations with biomarkers of ascending thoracic aortic aneurysm. Ann Thorac Surg. (2020) 110:1595–604. 10.1016/j.athoracsur.2020.03.017 32289298

[20] Bollache E Guzzardi DG Sattari S Olsen KE Di Martino ES Malaisrie SC. Aortic valve-mediated wall shear stress is heterogeneous and predicts regional aortic elastic fiber thinning in bicuspid aortic valve-associated aortopathy. J Thorac Cardiovasc Surg. (2018) 156:2112–20.e2. 10.1016/j.jtcvs.2018.05.095 30060930 PMC6242768

[21] Stemper BD Yoganandan N Stineman MR Gennarelli TA Baisden JL Pintar FA. Mechanics of fresh, refrigerated, and frozen arterial tissue. J Surg Res. (2007) 139:236–42. 10.1016/j.jss.2006.09.001 17303171

[22] O'Leary SA Doyle BJ McGloughlin TM. The impact of long term freezing on the mechanical properties of porcine aortic tissue. J Mech Behav Biomed Mater. (2014) 37:165–73. 10.1016/j.jmbbm.2014.04.015 24922621

[23] Sigaeva T Polzer S Vitasek R Di Martino ES. Effect of testing conditions on the mechanical response of aortic tissues from planar biaxial experiments: Loading protocol and specimen side. J Mech Behav Biomed Mater. (2020) 111:103882. 10.1016/j.jmbbm.2020.103882 32745968

[24] Chung J Lachapelle K Wener E Cartier R De Varennes B Fraser R. Energy loss, a novel biomechanical parameter, correlates with aortic aneurysm size and histopathologic findings. J Thorac Cardiovasc Surg. (2014) 148:1082–9. 10.1016/j.jtcvs.2014.06.021 25129601

[25] Schindelin J Arganda-Carreras I Frise E Kaynig V Longair M Pietzsch T. Fiji: an open-source platform for biological-image analysis. Nat Methods. (2012) 9:676–82. 10.1038/nmeth.2019 22743772 PMC3855844

[26] Di Martino ES Bohra A Vande Geest JP Gupta N Makaroun MS Vorp DA. Biomechanical properties of ruptured versus electively repaired abdominal aortic aneurysm wall tissue. J Vasc Surg. (2006) 43:570–6. 10.1016/j.jvs.2005.10.072 16520175

[27] Haller S Crawford J Courchaine K Bohannan C Landry G Moneta G. Intraluminal thrombus is associated with early rupture of abdominal aortic aneurysm. J Vasc Surg. (2018) 67:1051–8. 10.1016/j.jvs.2017.08.069 29141786

[28] Vorp D Lee P Wang D Makaroun M Nemoto E Ogawa S. Association of intraluminal thrombus in abdominal aortic aneurysm with local hypoxia and wall weakening. J Vasc Surg. (2001) 34:291–9. 10.1067/mva.2001.114813 11496282

[29] Kazi M Thyberg J Religa P Roy J Eriksson P Hedin U. Influence of intraluminal thrombus on structural and cellular composition of abdominal aortic aneurysm wall. J Vasc Surg. (2003) 38:1283–92. 10.1016/S0741-5214(03)00791-2 14681629

[30] Martufi G Satriano A Moore RD Vorp DA Di Martino ES. Local quantification of wall thickness and intraluminal thrombus offer insight into the mechanical properties of the aneurysmal aorta. Ann Biomed Eng. (2015) 43:1759–71. 10.1007/s10439-014-1222-2 25631202

[31] Hellenthal FAMVI Buurman WA Wodzig WKWH Schurink GWH. Biomarkers of AAA progression. Part 1: extracellular matrix degeneration. Nat Rev Cardiol. (2009) 6:464–74. 10.1038/nrcardio.2009.80 19468292

[32] Curci JA Liao S Huffman MD Shapiro SD Thompson RW. Expression and localization of macrophage elastase (matrix metalloproteinase-12) in abdominal aortic aneurysms. J Clin Invest. (1998) 102:1900–10. 10.1172/JCI2182 9835614 PMC509141

[33] Baxter B McGee G Shively V Drummond I Dixit S Yamauchi M. Elastin content, cross-links, and mRNA in normal and aneurysmal human aorta. J Vasc Surg. (1992) 16:192–200. 10.1016/0741-5214(92)90107-J 1495142

[34] Carmo M Colombo L Bruno A Corsi F Roncoroni L Cuttin M. Alteration of elastin, collagen and their cross-links in abdominal aortic aneurysms. Eur J Vasc Endovasc Surg. (2003) 23:543–9. 10.1053/ejvs.2002.1620 12093072

[35] Rizzo RJ McCarthy WJ Dixit SN Lilly MP Shively VP Flinn WR. Collagen types and matrix protein content in human abdominal aortic aneurysms. J Vasc Surg. (1989) 10:365–73. 10.1016/0741-5214(89)90409-6 2795760

[36] Sakalihasan N Heyeres A Nusgens BV Limet R Lapiére CM. Modifications of the extracellular matrix of aneurysmal abdominal aortas as a function of their size. Eur J Vasc Surg. (1993) 7:633–7. 10.1016/S0950-821X(05)80708-X 8270064

[37] Hurks R Pasterkamp G Vink A Hoefer I Bots M Van De Pavoordt H. Circumferential heterogeneity in the abdominal aortic aneurysm wall composition suggests lateral sides to be more rupture prone. J Vasc Surg. (2012) 55:203–9. 10.1016/j.jvs.2011.06.113 21944916

[38] Brown A Shi Y Marzo A Staicu C Valverde I Beerbaum P. Accuracy vs. computational time: translating aortic simulations to the clinic. J Biomech. (2012) 45:516–23. 10.1016/j.jbiomech.2011.11.041 22189248

[39] Vorp DA Schiro BJ Ehrlich MP Juvonen TS Ergin MA Griffith BP. Effect of aneurysm on the tensile strength and biomechanical behavior of the ascending thoracic aorta. Ann Thorac Surg. (2003) 75:1210–4. 10.1016/S0003-4975(02)04711-2 12683565

